# Supplementary material for: Learning in a sensory cortical microstimulation task is associated with elevated representational stability
Source: Nat Commun. 2023 Jun 29;14:3860. doi: 10.1038/s41467-023-39542-x (PMC10310840; doi:10.1038/s41467-023-39542-x)
Supplement: Supplementary file 1 — Supplementary Information [file 41467_2023_39542_MOESM1_ESM.pdf]

SUPPLEMENTARY INFORMATION

Animal ID	Sex	Age at training start (days)	Opsin+ cell count	Opsin− cell count	Learning status	Opsin expression
**293010**	M	75	775	2593	Learner	Yes
**293157**	M	96	718	2679	Learner	Yes
**293167**	M	66	486	2413	Learner	Yes
**295578**	M	77	624	2640	Learner	Yes
**295678**	M	83	836	1621	Learner	Yes
**010852**	M	87	717	1778	Learner	Yes
**293156**	M	107	725	2530	Non-learner	Yes
**294755**	M	90	766	2360	Non-learner	Yes
**295137**	M	74	863	2183	Non-learner	Yes
**295331**	M	83	693	2178	Non-learner	Yes
**010580**	M	92	681	1609	Non-learner	Yes
**297108**	M	67	N/A	N/A	N/A	No
**297109**	M	67	N/A	N/A	N/A	No
**297670**	M	58	N/A	N/A	N/A	No
**297023**	M	81	N/A	N/A	N/A	No
**297027**	F	82	N/A	N/A	N/A	No
**007417**	M	N/A	829	2731	N/A	Yes

Supplementary Table 1. **List of animals**. All mice were transgenic Ai162 X Slc17a7-Cre (Daigle et al., 2018), expressing GCaMP6s exclusively in excitatory neurons. Opsin non-expressing controls were only used in Figure 1. One animal (0074147) was never trained and used for calibration of photostimulation parameters in **Supplementary Fig. 3**.

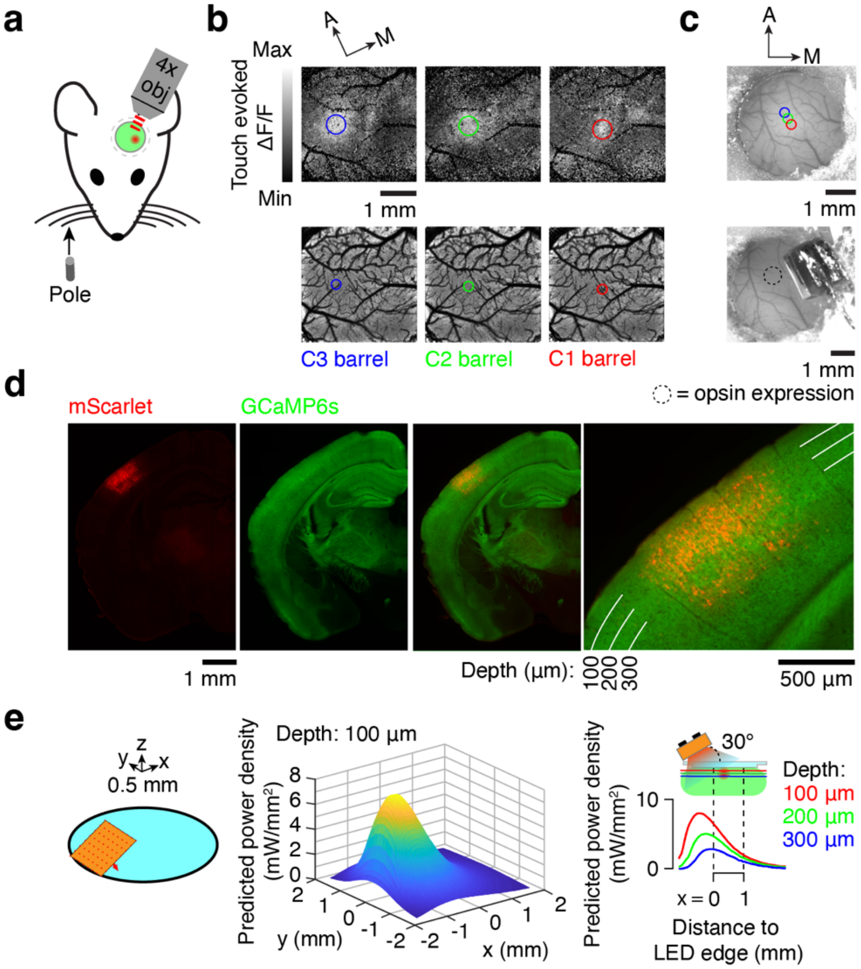

Supplementary Figure 1. Targeting single barrel for superficial opsin expression. **a.** Barrel mapping was conducted by driving a pole into whiskers C1-3 one at a time during widefield (4x) two-photon calcium imaging. **b.** Touch-evoked ΔF/F following whisker stimulation. Top, ΔF/F following touch by indicated whisker; bottom, image showing barrel locations and corresponding vasculature. **c.** Cranial window prior to LED placement (top) and following LED placement (bottom). Colored circles indicate the identified barrel locations from (B), and the dotted circle indicates the site of opsin expression. **d.** Opsin expression. Left to right: expression of mScarlet, the fluorophore co-expressed with ChRmine; expression of GCaMP6s in a coronal section; overlay of GCaMP6s and mScarlet; magnified overlay. **e.** Simulation of light propagation in the opsin-expressing tissue (Methods). Left, geometric model of the LED and cranial window; center, predicted power density surface at a depth of 100 µm in the brain; right, predicted power density at various depths in the brain and distances from the LED edge.

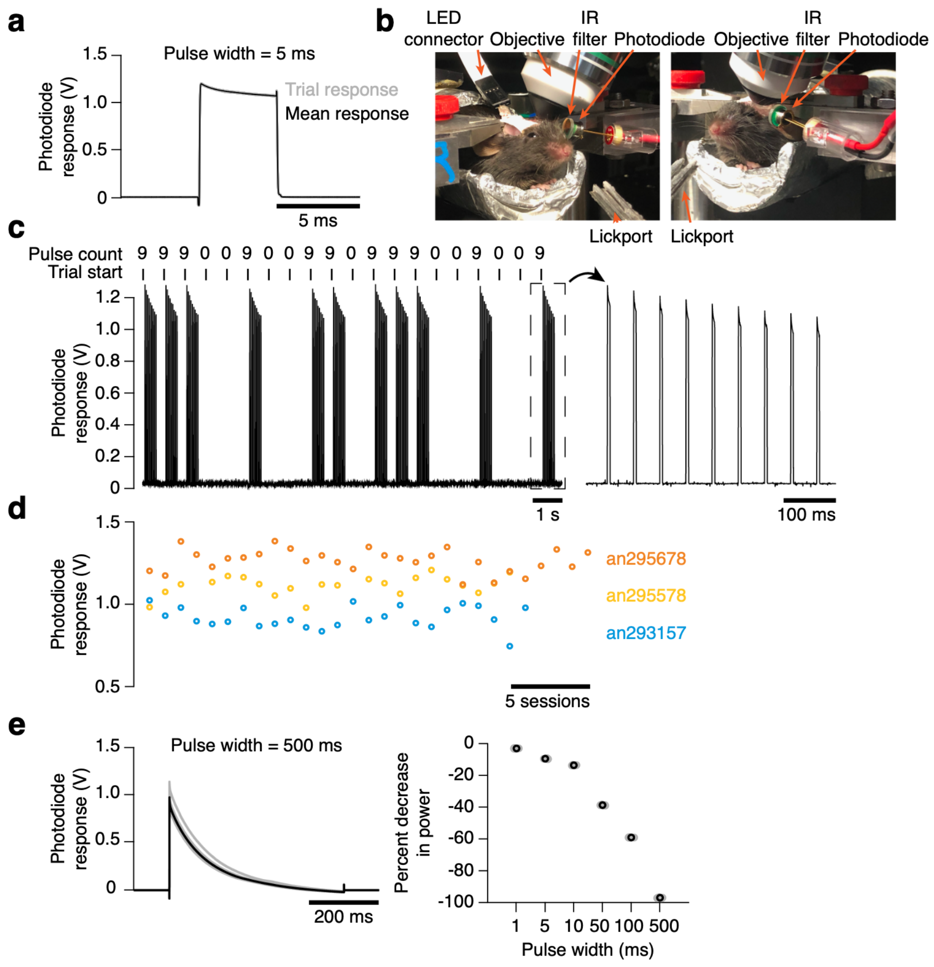

Supplementary Figure 2. LED stimulation is robust and reproducible. a. LED responses as measured by a standalone photodiode. 10 repetitions of a single 5 ms pulse at 100% power. Grey, individual repetitions; black, mean. **b.** Placement of photodiode used to measure LED output. **c.** Photodiode responses to LED stimulation across 20 consecutive trials in one behavioral session. **d.** Photodiode responses to 9 pulse trials across sessions for 3 animals. LED was tested for 10-20 trials at the conclusion of each behavioral session following removal of water for imaging objective. **e.** Photodiode responses to long duration pulses. Left, same as **a** for a 500 ms pulse; right, percent decrease in power between pulse start and pulse end for various pulse widths. Grey, individual repetitions; black, mean across repetitions.

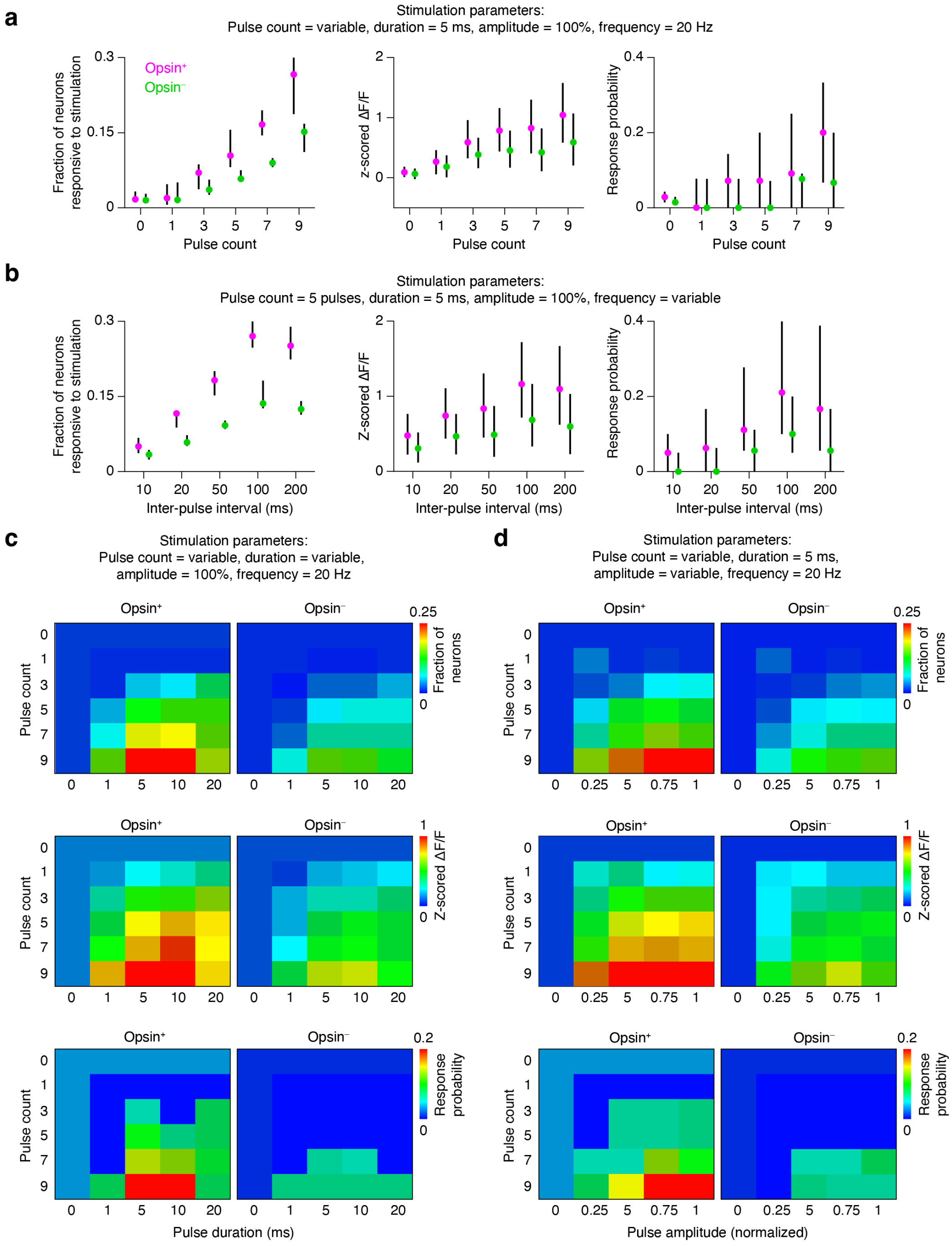

Supplementary Figure 3. Neural responsiveness varies as a function of stimulation parameters. a. Fraction of neurons responding (left), response amplitude (center), and response probability (right) as a function of pulse count for opsin-expressing (red) and opsin non-expressing (blue) neurons. Data are shown as median ± inter-quartile range for a single mouse that was awake but not performing a task (an007417, **Supplementary Table 1**). **b.** Same as **a** except as a function of inter-pulse interval. **c.** Fraction of neurons responding (top), response amplitude (middle), and response probability (bottom) as function of both pulse count and pulse duration. **d.** Same as **c** except for pulse count and pulse amplitude.

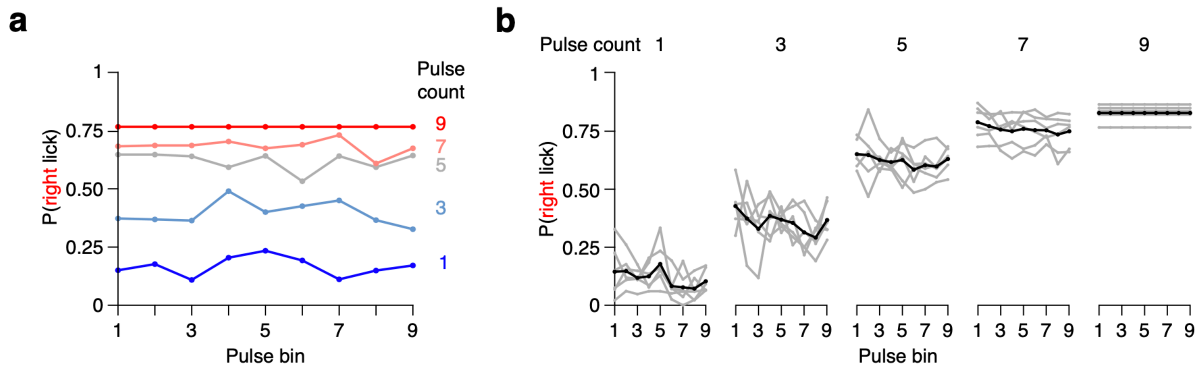

Supplementary Figure 4. Mice do not use pulse timing to solve the task. a. Right lick probability in each of the 9 possible pulse time bins for each pulse count in the final discrimination task for one animal. **b.** Same as **a** for all animals that attained the final task stage (n=6). Grey, individual animals; black, mean.

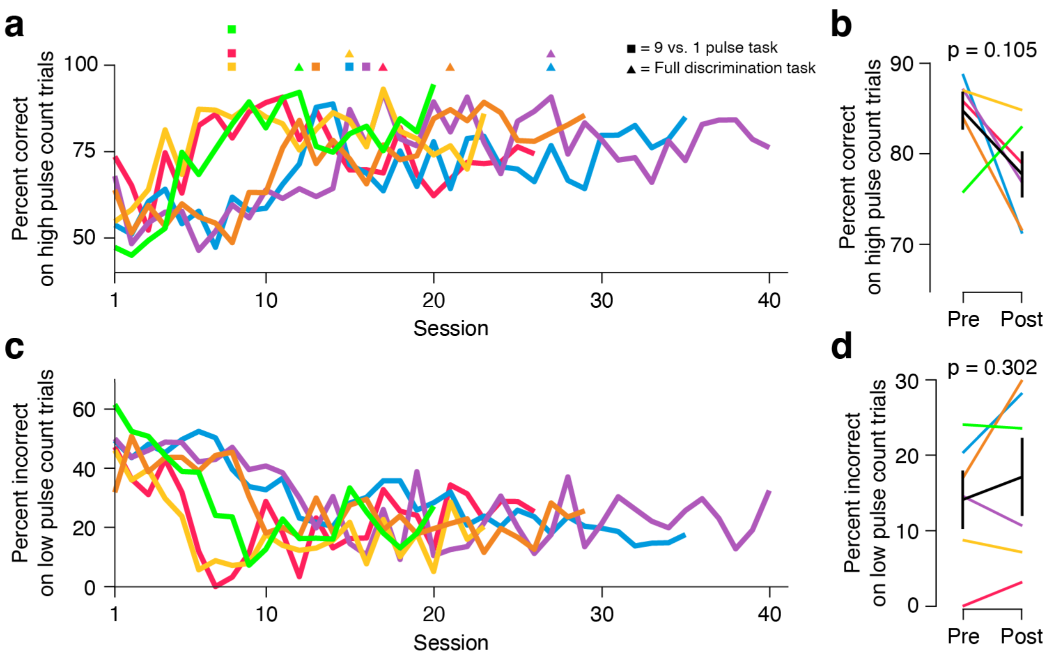

Supplementary Figure 5. Behavioral performance segregated by trial type. a. Percent correct on trials with high pulse count across all mice that learned the task. Shapes indicate transitions to successive task stages, colors indicate mice. **b.** Percent correct on high pulse count trials for sessions immediately prior to and after the transition from 9/0 to 9/1 task version (square in **a**). P-value reported for paired two-sided t-test. **c**, **d.** As in **a**, **b**, but instead showing percent incorrect on low pulse count trials (i.e., animal licked right on leftward trial).

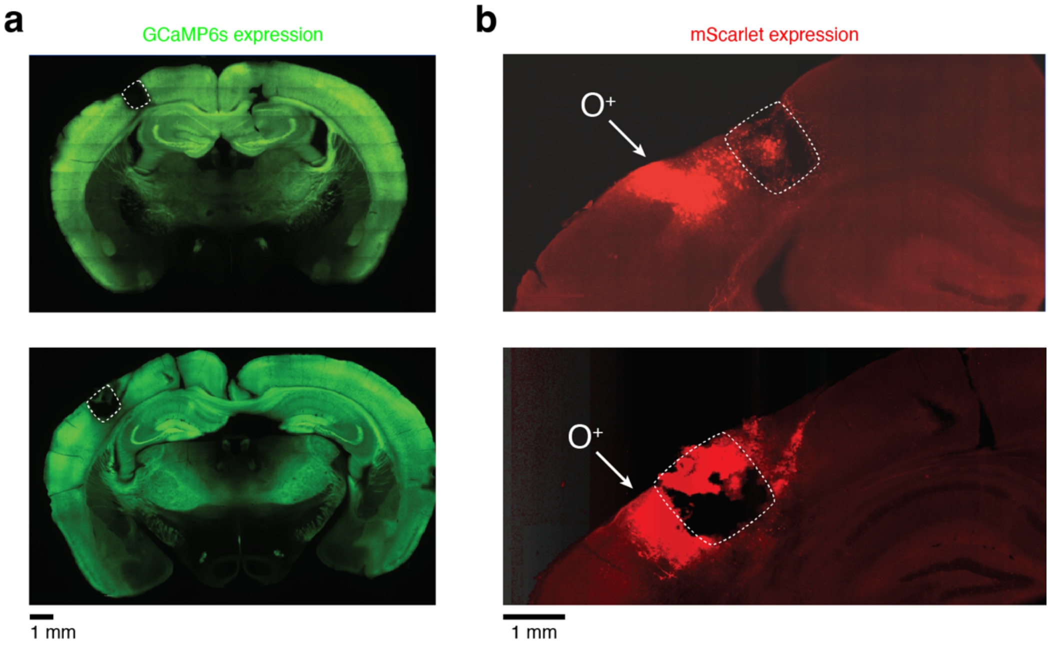

Supplementary Figure 6. Post-lesion histology. a. Coronal slices showing GCaMP6s expression and lesion sites in two example animals. **b.** mScarlet expression in the same two animals post-lesion. Remaining opsin-expressing neurons are indicated (O^+).

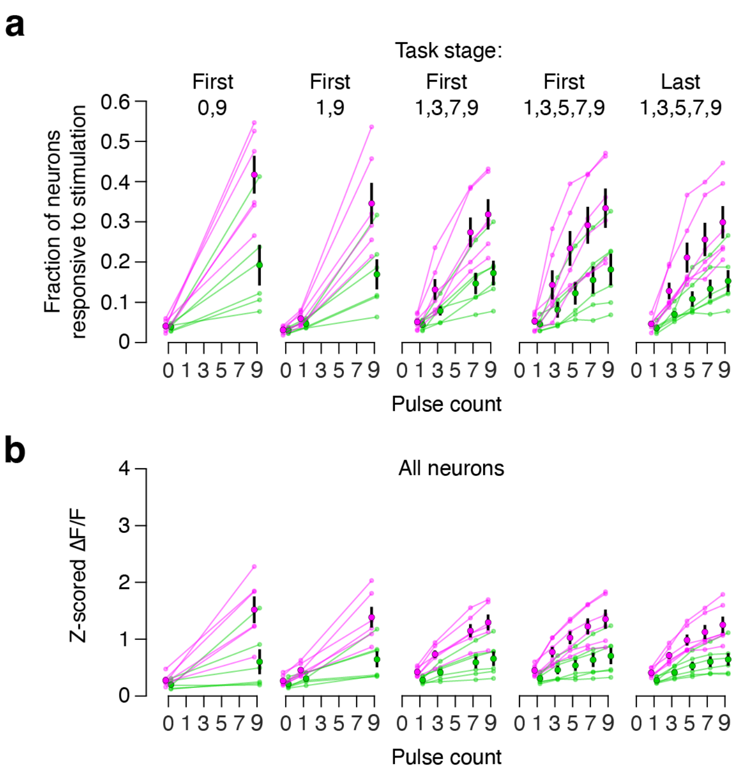

Supplementary Figure 7. Response to various pulse counts over the course of training. a. Fraction of neurons responsive to stimulation as a function of pulse count at different stages of training (small dot, median; large dot, grand mean; error bars, s.e.m.). Only animals that learned the task are included (n = 6). Individual lines show individual animals. Magenta, opsin-expressing; green, opsin non-expressing. **b.** As in **a**, but for response amplitude.

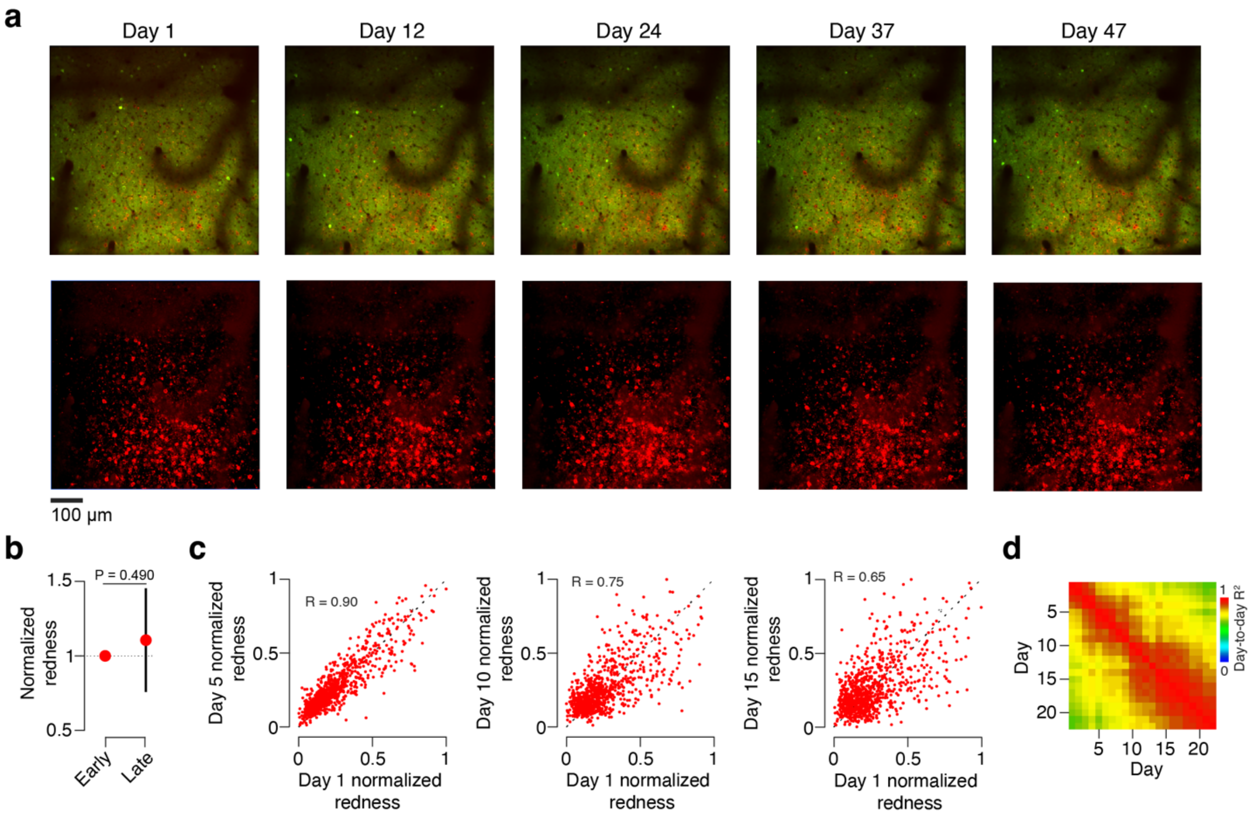

Supplementary Figure 8. Opsin expression over the course of learning. a. Example imaging plane across nearly 7 weeks. Top, GCaMP6s (green) and opsin (red) expression; bottom, linearly demixed red channel signal showing opsin expression. **b.** Mean redness for the earliest and latest sessions. Error bars, SEM. P-value given for two-sided paired t-test comparing early to late sessions (n = 6 mice, learners only). **c.** Relationship between redness on day 1 and redness on subsequent days for example animal. **d.** Day-to-day correlation of redness across all opsin-expressing neurons averaged across animals (n = 6 mice).

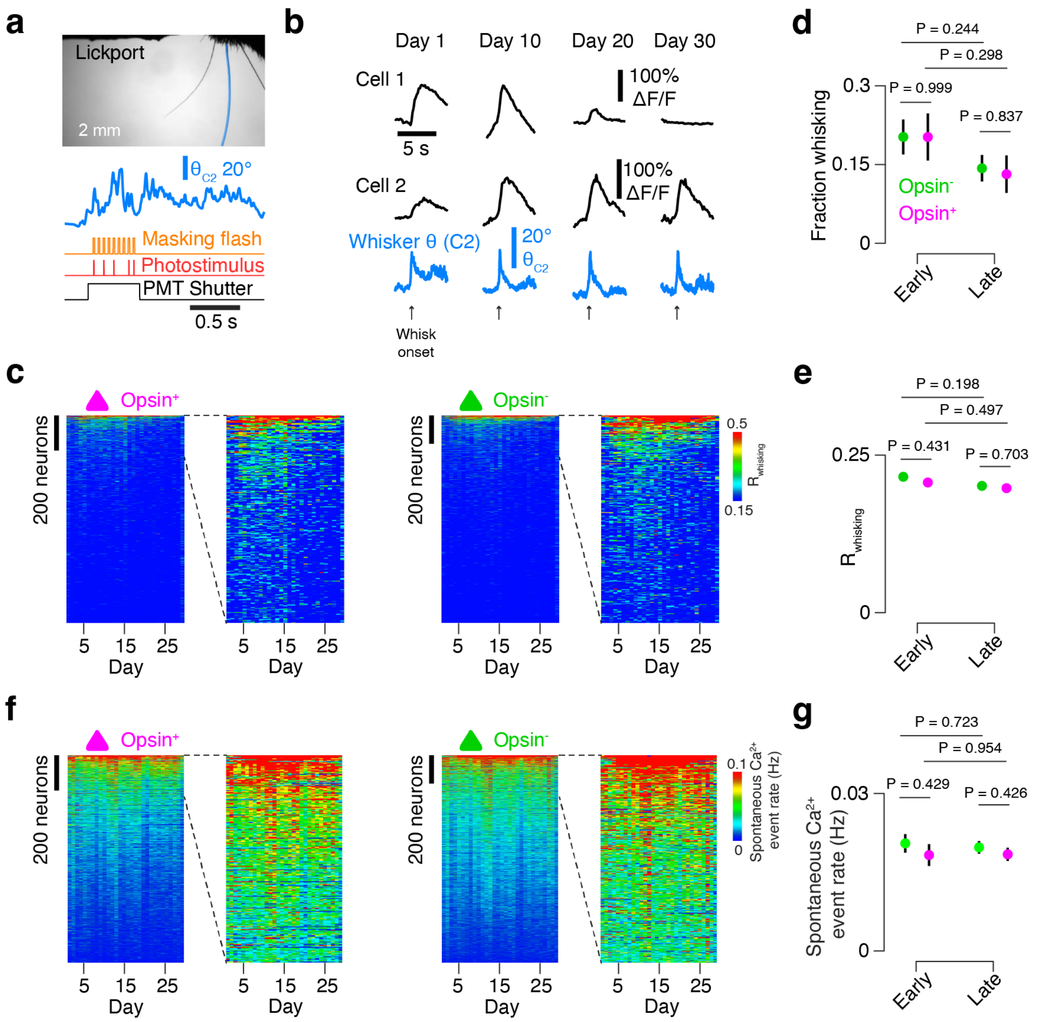

Supplementary Figure 9. Stability of natural whisking response and spontaneous activity. a. Example whisker videography (top) with C2 whisker outlined in blue. Bottom, whisker angle during an example trial, along with masking flash, photostimulus, and PMT shutter timing. **b.** ΔF/F in two example neurons (top, middle) and whisker angle (bottom, blue) across sessions averaged aligned to whisking onset for a given session. Lines show mean across multiple whisks. **c.** Whisking encoding score ($R_{whisking}$; Methods) across sessions for opsin-expressing (left, magenta) and opsin non-expressing neurons (right, green) from one example animal. **d.** Fraction of neurons classified as whisking cells on early and late sessions in mice that learned the task. Green, opsin non-expressing neurons; magenta: opsin-expressing neurons. Dot, mean; bar, SEM. Significance indicated for two-sided t-test comparing early to late values as well as opsin-expressing vs. opsin non-expressing (n = 6 mice, learners only). **e.** As in **d**, but for mean whisking encoding score. **f.** Spontaneous calcium event rate (Methods) across sessions from one example animal. **g.** Spontaneous calcium event rate on early and late sessions. Green, opsin non-expressing neurons; magenta: opsin-expressing neurons. Dot, mean; bar, SEM. Significance indicated for t-test comparing early to late values as well as opsin-expressing vs. opsin non-expressing (n = 6 mice).
